# Online Estimation of Manipulator Dynamics for Computed Torque Control of Robotic Systems

**DOI:** 10.3390/s25226831

**Published:** 2025-11-08

**Authors:** Hichem Kallel, Kamran Iqbal

**Affiliations:** 1 Mediterranean Institute of Technology, Southern Mediterranean University, Tunis 1053, Tunisia; hichem.kallel@medtech.tn; 2 School of Engineering and Engineering Technology, University of Arkansas at Little Rock, Little Rock, AR 72204, USA

**Keywords:** robotic systems, two-link robot manipulator, data-driven estimation and control, physics-informed neural networks

## Abstract

**Highlights:**

**What are the main findings?**

- This study presents a novel framework for the modeling and control of robotic systems based on data gathered from real-time sensors.
- We developed and tested a scheme to estimate robot dynamics online from the trajectory data gathered during robot movements.

**What is the implication of the main finding?**

- The proposed approach sidesteps the need for complete a priori knowledge of system parameters.
- Using this approach, robots with unmodeled dynamics can successfully operate in unknown environments.

**Abstract:**

Traditional control of robotic systems relies on the availability of an exact model, which assumes complete knowledge of the robot’s parameters and all dynamic effects. However, this idealized scenario rarely holds in practice, as real-world interactions introduce unpredictable environmental influences, friction, and edge effects. This paper presents a novel data-driven approach to modeling and estimating robot dynamics by leveraging data collected during the robot’s movements. The proposed method operates without prior knowledge of the system parameters, thereby addressing the limitations of conventional model-based control strategies in complex and uncertain environments. Our unified data-driven framework integrates classical control theory with modern machine learning techniques, including system identification, physics-informed neural networks (PINNs), and deep learning. We demonstrate its efficacy in the case of a two-link robotic manipulator that achieves superior trajectory tracking and robustness to unmodeled dynamics. The technique is modular and can be extended to manipulators with more joints.

## 1. Introduction

The control of robotic systems has traditionally relied on accurate and comprehensive mathematical models that describe a robot’s behavior under a wide range of operating conditions. In their seminal work, Slotine and Li [1] laid the foundation for adaptive control, which assumes complete knowledge of system parameters, including mass distribution, frictional forces, actuator dynamics, and other critical characteristics. However, in real-world applications, acquiring such complete and precise models is challenging due to the inherent complexity of robot–environment interactions. Factors such as external disturbances, sensor noise, and unmodeled dynamics (e.g., frictional nonlinearities, structural flexibilities, and edge effects) can significantly degrade the performance of traditional controllers as noted by Siciliano et al. [2].

Computed torque control (CTC), in particular, has emerged as one of the most effective model-based control strategies. Under the assumption of complete and precise modeling, this method demonstrates superior tracking accuracy and disturbance rejection. However, despite their effectiveness under ideal conditions, even minor inaccuracies or unmodeled dynamics can lead to performance degradation in model-based control. This has spurred research into data-driven approaches that aim to overcome these limitations by learning from real-time sensor data. Data-driven methods for CTC have gained traction as powerful alternatives to traditional model-based approaches in complex environments or modeling uncertainties. Unlike classical CTC, which relies on precise knowledge of the dynamic model of a robot (involving inertia, Coriolis, and gravity terms), data-driven techniques leverage machine learning to approximate these dynamics directly from sensor data, allowing robust control even with incomplete or inaccurate models. In particular, Gaussian Process Regression (GPR) [3] and deep neural networks [4] were employed to learn inverse dynamics models that replace or augment analytical formulations in CTC, thus enabling robust control even with incomplete or inaccurate models. Hybrid methods that combine physics-based models with data-driven corrections (e.g., residual learning) have shown improved generalization and safety. Overall, data-driven CTC methods offer increased flexibility, adaptability, and performance in modern robotic systems. Recent work by Kiumarsi et al. [5] and Chen et al. [6] has demonstrated that data-driven control strategies can adapt to changing environments and unmodeled dynamics, offering improved performance when the full model is not available.

Other recent approaches for robot control include deep reinforcement learning-based control strategies [7] and comprehensive surveys of data-driven robotic control methods [8]. Feedforward networks approximate nonlinear mappings from state control to state derivatives, serving as black-box dynamics models for model-based control or planning [9,10]. These approaches require training on a significant amount of data to generalize. Recurrent neural networks (RNN, LSTM, and GRU) capture temporal dependencies and partial observability, useful when dynamics depend on unmeasured states or previous history of control actions [11]. Non-parametric Gaussian processes (GPs) provide efficient probabilistic models that quantify uncertainty, supporting safe exploration and data-efficient learning [12,13]. These studies explore model-based policy search methods that incorporate a probabilistic dynamics model to express uncertainty. However, GP models are computationally expensive and scale poorly with data. Their application in robotics is limited to smooth and non-switching dynamics.

Physics-informed neural networks (PINNs) embed physical laws (expressed by partial differential equations, e.g., Euler–Lagrange equations) into the loss function or the network architecture, intending to improve data efficiency and generalization. PINNs have found significant applications in fluid dynamics, where they enable the accurate simulation of Navier–Stokes equations without requiring traditional mesh-based methods [14]. In biomedical engineering, PINNs assist in estimating parameters in models of blood flow and tissue mechanics, offering non-invasive diagnostic tools [15]. PINNs obviate the need for acquiring extensive experimental data for model training when the governing equations are known [16]. In the field of robotics, PINNs have been used for dynamic model prediction and parameter identification for collaborative robots [17]. In another study, PINN were employed to handle nonconservative effects for dynamic modeling and control of complex robotic systems [18]. By combining PINN with model-based controllers, precise control performance close to theoretical stability bounds was achieved. Recently, PINNs were used to build an efficient surrogate model that, when coupled with a nonlinear model predictive controller, enabled real-time optimization in legged locomotion [19].

Lagrangian and Hamiltonian neural networks enforce energy conservation constraints in physical systems. As a result, the Hamiltonian network trains faster and generalizes better than a regular neural network [11]. At the same time, learning physics models for model-based control requires robust extrapolation from fewer samples. A deep Lagrangian network can learn the equations of motion of a mechanical system efficiently while ensuring physical plausibility, and performs very well in robot tracking control [4]. However, Lagrangian networks only model conservative forces that do not include friction, damping, and contact effects.

This study presents a novel framework for the modeling and control of robotic systems based on data from real-time sensors to account for unmodeled dynamics. We describe how the parameters of the robot manipulator can be estimated online, followed by a CTC controller design based on the constructed model. The proposed approach eschews the need for complete a priori knowledge of system parameters, providing a viable solution for unknown environments where traditional control methods are not adequate.

Although numerous model-based and data-driven approaches have been proposed for robotic control, existing studies still face key limitations. Classical CTC assumes complete or partial knowledge of the robot dynamics, while purely data-driven models often require large offline datasets and lack physical consistency. Hybrid methods combining analytical and learning-based models partially address these issues but remain task-specific or limited in scope. To overcome these challenges, this paper introduces a unified data-driven framework for online estimation and control of robotic manipulators that merges the interpretability of physics-based models with the adaptability of modern learning. Our main contributions are as follows:

- Online estimation of gravity, Coriolis, frictional, and inertial effects directly from trajectory control data with no prior parameter knowledge.
- Use of PINNs for model learning to ensure physical consistency and stability.
- Integration of these estimates into a **CTC** structure for adaptive and robust tracking.

The rest of the paper is organized as follows. Control methodologies for robot manipulators are discussed in Section 2, followed by online estimation of the robot parameters in Section 3. Section 4 presents the results for the online estimation of the gravity, Coriolis, and inertial matrices, followed by a conclusion in Section 5.

## 2. Control Methodologies for Robot Manipulators

In this section, we first discuss the dynamic model of the robotic system and describe the CTC design, which will serve as a benchmark for performance. We then discuss how the data-driven control strategy estimates the dynamic parameters to enable real-time control of robotic systems.

### 2.1. Computed Torque Control (CTC)

CTC is a model-based scheme that leverages an accurate dynamic model of the robot to ensure stability and precision in tracking tasks. Consider the extended robot dynamics model given by

(1) $$M\left(q\right)\ddot{q}+C\left(q,\dot{q}\right)\dot{q}+g\left(q\right)+f\left(q,\dot{q}\right)=\tau ,$$

where:

- $q\in R^{n}$ is the vector of joint positions (where *n* is the number of joints).
- $M\left(q\right)\in R^{n\times n}$ is the symmetric positive definite inertia matrix.
- $C\left(q,\dot{q}\right)\in R^{n\times n}$ represents the Coriolis and centrifugal forces.
- $g\left(q\right)\in R^{n}$ denotes the gravitational forces.
- $f\left(q,\dot{q}\right)\in R^{n}$ models frictional forces, which can be expressed as a linear viscous term $F_{v}\dot{q}$ (with $F_{v}$ the positive definite viscous friction matrix) or via more complex models.
- $\tau \in R^{n}$ is the control input.

Feedback linearization uses the exact model to cancel nonlinear terms, transforming the closed-loop into a linear system controlled by proportional-derivative (PD) feedback controller gains. Lyapunov analysis ensures global asymptotic stability when the model is precise [20]. Assuming that M(q), $C(q,\dot{q})$, g(q), and $f(q,\dot{q})$ are exactly known, let $q_{d}\left(t\right)$ be the desired trajectory with derivatives ${\dot{q}}_{d}$ and ${\ddot{q}}_{d}$. Then, the traditional CTC law is defined as

(2) $$\tau =M\left(q\right)\left[{\ddot{q}}_{d}+K_{d}\left({\dot{q}}_{d}-\dot{q}\right)+K_{p}\left(q_{d}-q\right)\right]+C\left(q,\dot{q}\right)\dot{q}+g\left(q\right)+f\left(q,\dot{q}\right),$$

where $K_{p}$ and $K_{d}$ are positive definite gain matrices and, $q_{d}$, ${\dot{q}}_{d}$, and ${\ddot{q}}_{d}$ denote the desired joint position, velocity, and acceleration vectors, respectively. Define the tracking errors as

(3) $$e=q_{d}-q\;\mathrm{and}\;\dot{e}={\dot{q}}_{d}-\dot{q}.$$

The Lyapunov method is used to ensure stability. A Lyapunov function candidate is chosen as

(4) $$V\left(e,\dot{e}\right)=\frac{1}{2}\;{\dot{e}}^{T}M\left(q\right)\dot{e}+\frac{1}{2}\;e^{T}K_{p}e.$$

Taking the time derivative of *V* and substituting the closed-loop dynamics from (1) and (2), one can show that

(5) $$\dot{V}=-\;{\dot{e}}^{T}K_{d}\;\dot{e},$$

This guarantees that the tracking error converges to zero globally (or remains bounded under bounded disturbances), ensuring the stability of the closed-loop system.

### 2.2. Data-Driven Control

In the data-driven approach, the exact dynamics matrices and vectors are replaced by their estimates, which are learned from the sensor data. In particular, the following robot parameters are replaced by their predicted values:

- $\hat{M}\left(q\right)$: the estimated inertia matrix.
- $\hat{C}\left(q,\dot{q}\right)$: the estimated Coriolis and centrifugal force matrix.
- $\hat{g}\left(q\right)$: the estimated gravitational forces.
- $\hat{f}\left(q,\dot{q}\right)$: the estimated frictional forces.

Thus, the data-driven computed torque control law is formulated as

(6) $$\tau =\hat{M}\left(q\right)\left[{\ddot{q}}_{d}+K_{d}\left({\dot{q}}_{d}-\dot{q}\right)+K_{p}\left(q_{d}-q\right)\right]+\hat{C}\left(q,\dot{q}\right)\;\dot{q}+\hat{g}\left(q\right)+\hat{f}\left(q,\dot{q}\right),$$

where $q_{d}$, ${\dot{q}}_{d}$, and ${\ddot{q}}_{d}$ are the desired position, velocity, and acceleration trajectories, respectively, and $K_{p}$, $K_{d}$ are positive definite gain matrices. After learning the parameter values during training, these predictions enable the controller to adaptively compensate for the unmodeled dynamics and disturbances present in the real system.

#### Stability with Estimated Dynamics

In order to determine the stability, we denote the modeling errors by

$$\Delta M=M-\hat{M},\;\Delta C=C-\hat{C},\;\Delta g=g-\hat{g},\;\Delta f=f-\hat{f},$$

and the lumped uncertainty as

(7) $$d\left(q,\dot{q},{\ddot{q}}_{d},e,\dot{e}\right)≜\Delta M\left({\ddot{q}}_{d}+K_{d}\dot{e}+K_{p}e\right)+\Delta C\;\dot{q}+\Delta g+\Delta f.$$

We enforce the standard structural assumptions and robot properties on the following estimates:

(i) $\hat{M}\left(q\right)=\hat{M}{\left(q\right)}^{⊤}≻0$ (e.g., by symmetrization and eigenvalue flooring);
(ii) $\dot{\hat{M}}\left(q\right)-2\hat{C}\left(q,\dot{q}\right)$ is skew-symmetric (e.g., $\hat{C}$ built from Christoffel symbols of $\hat{M}$);
(iii) $\hat{f}\left(q,\dot{q}\right)$ is passive (e.g., viscous term with ${\hat{F}}_{v}⪰0$).

These constraints can be enforced by adding projection layers or post-processing of the learned maps.

To assess stability, we consider the Lyapunov function

(8) $$V\left(e,\dot{e}\right)=\frac{1}{2}{\dot{e}}^{⊤}\hat{M}\left(q\right)\;\dot{e}+\frac{1}{2}e^{⊤}K_{p}e.$$

Using the closed-loop dynamics, standard manipulator identities, and the properties above, one obtains

(9) $$\dot{V}=-{\dot{e}}^{⊤}K_{d}\dot{e}+{\dot{e}}^{⊤}d\left(q,\dot{q},{\ddot{q}}_{d},e,\dot{e}\right).$$

If the lumped uncertainty is bounded as $∥d\left(\cdot \right)∥\leq d_{\max}$ on the operating set (which can be estimated from validation errors of $\hat{M},\hat{C},\hat{g},\hat{f}$), then

(10) $$\dot{V}\leq -\lambda_{\min}\left(K_{d}\right){∥\dot{e}∥}^{2}+∥\dot{e}∥\;d_{\max}.$$

This inequality implies that the tracking error remains uniformly ultimately bounded (UUB) [20]. Increasing the damping gain $K_{d}$ or improving the accuracy of the estimate (reducing the $d_{max}$) tightens this bound. The robot trajectory remains stable and converges to a small neighborhood around the desired path. When the estimation error tends to zero, the controller recovers asymptotic stability, identical to the ideal computed torque case.

## 3. Online Estimation of Model Parameters

In this section, we describe how the various parameters in the robot model, that is, the inertial, Coriolis, friction, and gravity parameters, can be estimated by recording robot trajectories in real-time.

### 3.1. Estimation of Gravity

To estimate the gravitational torques g(q) in the robot dynamics, we exploit the fact that when the robot is in a steady state, the measured joint velocity $\dot{q}$ and joint acceleration $\ddot{q}$ are zero. Under these conditions, the dynamic model simplifies considerably. In particular, if the robot is held in a fixed position by a controller, the inertial term $M\left(q\right)\ddot{q}$ and the Coriolis/centrifugal term $C\left(q,\dot{q}\right)\dot{q}$ vanish, and friction effects are minimal or can be assumed negligible. Thus, in steady state, the applied control torque is primarily used to balance the gravitational torque, i.e.,

(11) τ=g(q).

To generate the required data for the estimation of gravity, we use a simple PD control law. In this setup, the desired velocity and acceleration are set to zero. The PD control law for each joint is given by

(12) $$\tau_{PD}=K_{p}\left(q_{d}-q\right)-K_{d}\dot{q},$$

where $K_{p}$ and $K_{d}$ are positive definite gain matrices, $q_{d}$ is the desired final position.

During the experiment, the robot is controlled using this PD law. Once it reaches a steady state (that is, when $\dot{q}\approx 0$ and $q\approx q_{d}$), the applied torque $\tau_{PD}$ compensates primarily for gravity. At this point, the joint positions *q* and the corresponding control torque $\tau_{PD}$ are recorded. Although the final position may not exactly match $q_{d}$, it still provides an acceptable data point. Repeating this process in various static positions in the workspace yields a set of data points $q,\tau_{PD}$.

The gravity vector $\hat{g}\left(q\right)$ is estimated using a Multilayer Perceptron (MLP) Regressor. This model learns the nonlinear mapping from the joint position to the steady-state torque. The MLP Regressor utilizes fully connected layers. To optimize performance and ensure robustness, the model’s architecture and learning parameters were selected via Randomized Search Cross-Validation (RandomizedSearchCV) [21]. The search space included varying the number of layers/neurons (e.g., (100,100), (200,100)), activation functions (‘relu’, ‘tanh’), L2 regularization (α), and initial learning rate. The best model parameters found are summarized below:

- **Model Type**: MLP Regressor.
- **Optimization**: Adam solver, with a maximum of 5000 iterations.
- **Best Architecture**: hidden layer size = (100, 100), with  relu activation.
- **Regularization**: α = 0.001

Before training, both the input positions q and the target torques $\tau_{PD}$ were standardized using a **StandardScaler**. This crucial preprocessing step ensures that all features have zero mean and unit variance, accelerating the convergence and improving the numerical stability during gradient descent optimization.

#### Stability Considerations for Gravity Estimation

For the robot manipulator with PD control with no gravity compensation, the Lyapunov function is typically defined as

(13) $$V=\frac{1}{2}{\dot{q}}^{⊤}M\left(q\right)\dot{q}+\frac{1}{2}{\left(q_{d}-q\right)}^{⊤}K_{p}\left(q_{d}-q\right).$$

This Lyapunov function *V* is always positive (except at equilibrium when it is zero), and its time derivative is given by

(14) $$\dot{V}=-{\dot{q}}^{⊤}K_{d}\dot{q}-{\dot{q}}^{⊤}F_{v}\dot{q}\leq 0,$$

which is non-positive, indicating that the total “energy” of the system decreases over time. Hence, the closed-loop system is stable under PD control without gravity compensation. Although the PD controller ensures stability, it exhibits a steady-state offset due to uncompensated gravitational torques. This behavior is consistent with the data generation objective, which focuses on the static equilibrium states of the manipulator.

### 3.2. Estimation of Combined Coriolis and Friction Effects (C + f)

The combined effects of Coriolis, centrifugal, and friction forces are denoted as

(15) $$C\left(q,\dot{q}\right)\;\dot{q}+f\left(q,\dot{q}\right).$$

To estimate these forces, we take advantage of the data collected during robot motion at a constant speed. Under such conditions, the acceleration is approximately zero ($\ddot{q}\approx 0$), so the dynamic model in (1) simplifies to

(16) $$\tau \approx g\left(q\right)+C\left(q,\dot{q}\right)\;\dot{q}+f\left(q,\dot{q}\right).$$

Assuming that external disturbances d(t) are negligible or have been filtered out, and using our previously estimated gravity model $\hat{g}\left(q\right)$, we can isolate the combined term by subtracting the estimated gravitational component from the measured torque:

(17) $$\tau -\hat{g}\left(q\right)\approx C\left(q,\dot{q}\right)\;\dot{q}+f\left(q,\dot{q}\right).$$

To generate a constant-speed motion trajectory, we consider implementing the control law given by a PID Position Controller with Estimated Gravity Compensation:

(18) $$\tau =\hat{g}\left(q\right)+K_{d}\left({\dot{q}}_{d}-\dot{q}\right)+K_{p}\left(q_{d}-q\right)+K_{i}\;\int \left(q_{d}-q\right)\;dt,$$

where $K_{i}$ is a positive definite gain matrix for the integral action. For constant-speed motion, the desired trajectory is defined as ${\dot{q}}_{d}=v_{\mathrm{const}}$, and $q_{d}\left(t\right)$ is its integral. This control law ensures that the torque τ compensates for gravity while the integral term generates the necessary steady-state torque to overcome unmodeled Coriolis and friction effects, driving the position and velocity errors to zero. Once the control action is applied at a constant speed, the term $\tau -\hat{g}\left(q\right)$ effectively captures the combined dynamics:

(19) $$M\left(q\right)\ddot{q}+C\left(q,\dot{q}\right)\;\dot{q}+f\left(q,\dot{q}\right)-\tilde{g}\left(q\right),$$

where $\tilde{g}\left(q\right)=g\left(q\right)-\hat{g}\left(q\right)$ denotes the residual gravity estimation error. By collecting data during these stable, constant-speed movements, we record the joint positions *q*, the joint velocities $\dot{q}$, and compute the total residual torque $\tau -\hat{g}\left(q\right)$, which is dominated by the unmodeled dynamic effects. This yields a dataset $\left\{\left(q,\dot{q},\tau -\hat{g}\left(q\right)\right)\right\}$, which is used to train a regression model to predict the combined Coriolis and friction terms based on *q* and $\dot{q}$.

#### Stability Considerations for Coriolis and Friction Effects

The controller incorporates the learned gravity term and is expressed as

$$\tau =\hat{g}\left(q\right)+K_{d}\left({\dot{q}}_{d}-\dot{q}\right)+K_{p}\left(q_{d}-q\right)+K_{i}\;\int \left(q_{d}-q\right)\;dt.$$

Defining the position error $e=q_{d}-q$, velocity error $\dot{e}={\dot{q}}_{d}-\dot{q}$, and integral error z=∫edt, the closed-loop dynamics can be written as

$$M\left(q\right)\ddot{e}+K_{d}\dot{e}+K_{p}e+K_{i}z=d\left(q,\dot{q},e,\dot{e}\right),$$

where $d(q,\dot{q},e,\dot{e})$ contains all uncompensated terms, including Coriolis, friction, and residual gravity. Consider the Lyapunov function candidate

$$V\left(e,\dot{e},z\right)\;=\;\frac{1}{2}\;{\dot{e}}^{⊤}M\left(q\right)\;\dot{e}\;+\;\frac{1}{2}\;e^{⊤}K_{p}\;e\;+\;\frac{1}{2}\;z^{⊤}K_{i}\;z,$$

Differentiating *V* and substituting the closed-loop error dynamics together with the skew-symmetry property $\dot{M}\left(q\right)-2C\left(q,\dot{q}\right)$ being skew-symmetric (so that ${\dot{e}}^{⊤}\left(\dot{M}-2C\right)\dot{e}=0$), yields

$$\dot{V}\;=\;-\;{\dot{e}}^{⊤}K_{d}\;\dot{e}\;+\;{\dot{e}}^{⊤}d\left(q,\dot{q},e,\dot{e}\right).$$

If $∥d\left(q,\dot{q},e,\dot{e}\right)∥\leq D_{\max}$ on the operating set, then This establishes Uniform Ultimate Boundedness [20] of the tracking errors, with the ultimate bound tightened by larger $K_{d}$ or smaller $D_{\max}$. Hence, the closed-loop system remains stable, and the joint velocities converge to ${\dot{q}}_{d}$, enabling reliable collection of torque data.

### 3.3. Estimation of the Inertia Matrix

Once the gravitational, Coriolis, and frictional torques have been factored out, the inertial torques can be estimated during the normal operation of the robot, i.e., sensor data obtained during joint acceleration and deceleration.

The training data for the physics-informed neural network (PINN) was generated through a high-fidelity simulation of a two-link robotic manipulator under closed-loop control. This section details the key components of the data generation pipeline.

#### 3.3.1. Closed-Loop Trajectory Control Design

During the inertia estimation phase, the torque applied to the manipulator is generated by the controller, which combines the learned dynamic model with feedback regulation. The control law is expressed as

(20) $$\tau =\hat{C}\left(q,\dot{q}\right)\dot{q}+\hat{f}\left(q,\dot{q}\right)+\hat{g}\left(q\right)+K_{d}\left({\dot{q}}_{d}-\dot{q}\right)+K_{p}\left(q_{d}-q\right)+K_{i}\;\int \left(q_{d}-q\right)\;dt.$$

Here, the feedforward terms $\hat{C}\left(q,\dot{q}\right)\dot{q}$, $\hat{g}\left(q\right)$, and $\hat{f}\left(q,\dot{q}\right)$ compensate for the Coriolis, gravitational, and frictional effects, while the PID feedback terms ensure a stable and sufficiently excited motion. At each sampling instant, the applied control torque τ and the joint states $\left(q,\dot{q},\ddot{q}\right)$ are recorded.

To isolate the inertial component of the dynamics, the predicted gravity, Coriolis, and friction terms are subtracted from the total applied torque, yielding the residual torque

(21) $$\tau_{\mathrm{res}}=\tau -\hat{C}\left(q,\dot{q}\right)\dot{q}-\hat{g}\left(q\right)-\hat{f}\left(q,\dot{q}\right).$$

This residual term corresponds to the torque portion responsible for generating joint acceleration, i.e., the inertial contribution $M\left(q\right)\ddot{q}$, up to modeling and estimation errors. Physically, $\tau_{\mathrm{res}}$ represents the effective torque that drives acceleration once all other dynamic effects have been compensated by the learned models.

Consequently, each recorded sample $\left(q,\dot{q},\ddot{q},\tau_{\mathrm{res}}\right)$ captures the instantaneous relationship between joint accelerations and the corresponding inertial torque. The complete dataset is defined as

(22) $$D={\left\{\left(q^{\left(i\right)},{\dot{q}}^{\left(i\right)},{\ddot{q}}^{\left(i\right)},\tau_{\mathrm{res}}^{\left(i\right)}\right)\right\}}_{i=1}^{N},$$

where *N* denotes the total number of recorded data samples collected during all simulation runs. This dataset serves as the basis for learning the inertia matrix M(q) through the physics-informed neural network. The proposed approach ensures that the collected data directly represent the inertial dynamics of the manipulator, while the feedback controller maintains stability and provides sufficient excitation for accurate parameter identification.

#### 3.3.2. Physics-Informed Inertia Matrix Estimation

The inertia matrix M(q) is a fundamental component of robotic dynamics that encodes the mass distribution and coupling effects between joints. Traditional methods of estimating M(q) require precise knowledge of the robot’s physical parameters, which are often unavailable in practice. We present a data-driven approach using neural networks to learn the inertia matrix directly from operational data.

This study presents a PINN approach for learning the inertia matrix of a robotic manipulator. The method combines data-driven learning with fundamental physics constraints to ensure physically consistent predictions. By embedding the structure of the Euler–Lagrange equations into the neural network architecture and training process, we achieve accurate parameter estimation while maintaining physical plausibility. We implement a feedforward neural network $f_{\theta }:R^{n}\to R^{n(n+1)/2}$ that maps joint configurations to the unique elements of the symmetric inertia matrix:

(23) $$f_{\theta }\left(q\right)=\left[{\hat{M}}_{11},{\hat{M}}_{12},\ldots ,{\hat{M}}_{1n},{\hat{M}}_{22},\ldots ,{\hat{M}}_{nn}\right].$$

The inertia matrix is then constructed to ensure physical consistency:

(24) $$M\left(q\right)=\left[\begin{array}{cccc} e^{{\hat{M}}_{11}} & {\hat{M}}_{12} & \ldots & {\hat{M}}_{1n}\\ {\hat{M}}_{12} & e^{{\hat{M}}_{22}} & \ldots & {\hat{M}}_{2n}\\ \vdots & \vdots & \ddots & \vdots \\ {\hat{M}}_{1n} & {\hat{M}}_{2n} & \ldots & e^{{\hat{M}}_{nn}} \end{array}\right].$$

This parameterization guarantees the following:

- Positive definiteness through exponential terms on the diagonal.
- Symmetry by construction.
- Continuous and differentiable dependence on the joint configuration *q*

The PINN architecture predicts the unique elements of the inertia matrix. The PINN training incorporates two loss terms:

1. **Data fitting loss**:
  (25) $$L_{\mathrm{mse}}={∥\tau_{\mathrm{res}}-\hat{M}\left(q\right)\ddot{q}∥}^{2}$$
2. **Physics consistency loss**:

(26) $$L_{\mathrm{phys}}={\left[{\dot{q}}^{T}\left(\frac{d\hat{M}}{dt}-2C\right)\dot{q}\right]}^{2}$$

where $\frac{d\hat{M}}{dt}$ is computed via automatic differentiation. The  Jacobian computation is achieved via TensorFlow’s automatic differentiation [22]. The total loss is

(27) $$L_{\mathrm{total}}=L_{\mathrm{mse}}+\lambda_{\mathrm{phys}}L_{\mathrm{phys}}$$

where $\lambda_{\mathrm{phys}}$ is the tuning scalar hyperparameter coefficient that balances how much emphasis is placed on the physics-consistency term versus the purely data-fitting term in the total loss.

#### 3.3.3. Stability Considerations for Estimation of Inertial Dynamics

Using the robot dynamics and defining the position error $e=q_{d}-q$, the velocity error $\dot{e}={\dot{q}}_{d}-\dot{q}$, and the integral of the position error z=∫edt, The closed-loop error dynamics become

(28) $$M\left(q\right)\ddot{e}+C\left(q,\dot{q}\right)\dot{e}+K_{d}\dot{e}+K_{p}e+K_{i}z=\Delta ,$$

where $\Delta =\left(C-\hat{C}\right)\dot{q}+\left(g-\hat{g}\right)+\left(f-\hat{f}\right)$ represents the bounded modeling error, with $∥\Delta ∥\leq D_{\max}$. Using the Lyapunov candidate function

(29) $$V=\frac{1}{2}{\dot{e}}^{⊤}M\left(q\right)\dot{e}+\frac{1}{2}e^{⊤}K_{p}e+\frac{1}{2}z^{⊤}K_{i}z,$$

and the skew-symmetry property $\dot{M}\left(q\right)-2C\left(q,\dot{q}\right)$, the time derivative of *V* satisfies

(30) $$\dot{V}\leq -\lambda_{\min}\left(K_{d}\right){∥\dot{e}∥}^{2}+∥\dot{e}∥D_{\max}.$$

where $\lambda_{\min}\left(K_{d}\right)$ denotes the smallest eigenvalue of the positive-definite gain matrix $K_{d}$, representing the minimum damping level in the control system. Hence, the tracking error is UUB [20]. Increasing the damping gain $K_{d}$ or improving the accuracy of the learned models (reducing $D_{\max}$) tightens this bound. This controller ensures stable tracking performance without requiring knowledge of the inertia matrix, while still benefiting from learned compensation of gravitational, Coriolis, and frictional effects.

## 4. Results and Discussion

All simulations were conducted using a planar two-link robotic manipulator modeled with standard rigid-body dynamics. The mechanical and inertial parameters of each link are listed in Table 1. These values are consistent with benchmark models widely used in robotic control studies.

The controllers used during data generation are summarized in Table 2. The proportional–derivative (PD) controller was employed for static equilibrium tests to estimate gravitational torques, whereas the velocity–integral controller was used for constant-velocity experiments to isolate Coriolis and frictional effects. The computed-torque controller, incorporating the learned dynamics, was used for trajectory-tracking experiments.

Simulation results for online parameter estimation in the case of a two-link robotic manipulator are presented below, starting with online estimation of gravitational torques, followed by the estimation of Coriolis and inertia matrices.

### 4.1. Estimation of Gravitational Torques

**Figure 1:**
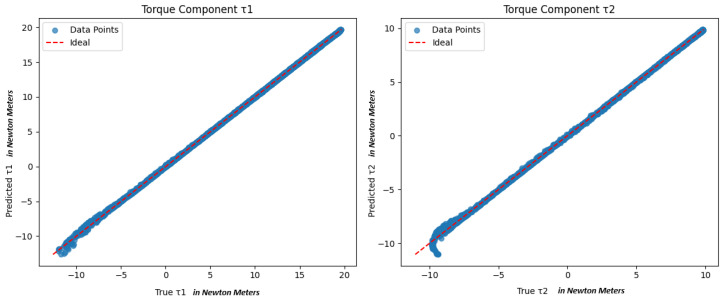
Comparison of true vs. predicted torque components $\tau_{1}$ and $\tau_{2}$ all in Nm. Red dashed line is the ideal y=x line.

We conducted 20,000 simulations to construct the dataset $\left\{\left(q,\tau_{PD}\right)\right\}$. Eighty percent of the dataset was used to train the optimized model, and a dedicated 20% held-out test set was used to assess its generalization capability across the operational space. Figure 1 illustrates the PD control true robot joint torques $\tau_{PD}=\left[\mathrm{True}\;\tau_{1},\mathrm{True}\;\tau_{2}\right]$ versus the predicted joint torques $\hat{g}\left(q\right)=\left[\mathrm{Predicted}\;\tau_{1},\mathrm{Predicted}\;\tau_{2}\right]$ when the manipulator reached various static configurations. The trained multi-layer perceptron (MLP) model predicts the static torques required to hold a two-link robotic arm at desired joint angles with very high accuracy, as evidenced by a mean squared error (MSE) of approximately 0.01048 (Nm)^2^ and an $R^{2}$ value of about 0.9997 on the test set. This near-perfect alignment is also evident in the scatter plots of predicted versus true torque components, where the data points lie almost exactly on the ideal y=x line. The simulations rely on a PD controller to drive the arm to various final positions within [−π/2,π/2] for each joint, effectively sampling a wide range of configurations. Because the final torques in this setup largely compensate for gravity (with a small PD offset), the collected dataset captures the essential information for learning the torque–angle relationship.

The sample size of 20,000 simulated configurations was determined based on the model’s complexity and empirical convergence analysis. Following standard neural network heuristics (N≥10W, where *W* denotes the number of trainable parameters), the adopted two-hidden-layer MLP with approximately 12,000 parameters would nominally require on the order of $10^{5}$ samples for exhaustive coverage of the configuration space. However, due to the smoothness and low stochasticity of the simulated torque–angle mapping, 20,000 samples were experimentally found to ensure stable generalization with no significant accuracy gain beyond this threshold. Model hyperparameters were tuned using 5-fold cross-validation within the RandomizedSearchCV [21] framework to guarantee robust parameter selection. All simulations were performed in Python 3.10 on Google Colab using a fourth-order Runge–Kutta integrator for trajectory computation.

### 4.2. Estimation of Coriolis and Friction Effects

A total of 44,548 samples were generated through numerical simulations of a two-link robotic manipulator operating under constant-velocity conditions. Each simulation lasted 10 s with a sampling period of 0.01 s, producing approximately 1000 time steps per run. A total of 3000 simulations were executed in parallel, each with randomly initialized joint angles and velocities, and distinct desired joint velocity vectors ${\dot{q}}_{d}$ drawn uniformly within [0.3,0.7] rad/s. At each simulation step where the velocity tracking error satisfied $∥\dot{q}-{\dot{q}}_{d}∥<10^{-3}$, a data record was saved. The collected dataset contains six columns representing both the robot’s configuration and control signals:

$$[q_{1},q_{2},{\dot{q}}_{1},{\dot{q}}_{2},\tau_{1},\tau_{2}].$$

Here, $\left(q_{1},q_{2}\right)$ are the joint positions, $\left({\dot{q}}_{1},{\dot{q}}_{2}\right)$ are the corresponding joint velocities, and $\tau =[\tau_{1},\tau_{2}]$ denotes the total control torques computed by the velocity-integral controller that includes proportional and integral feedback terms, as well as gravity compensation through the pre-trained model $\hat{g}\left(q\right)$. The resulting dataset, consisting of 44,548 samples and 6 features, provides a rich and diverse collection of robot motion and control data suitable for learning dynamic models such as the Coriolis and friction components. To isolate the Coriolis and friction term, the exact combined term, $C_{\mathrm{exact}}$, was obtained by subtracting the predicted gravitational torque, $\hat{g}\left(q\right)$, from the total measured torque τ as expressed by

$$C_{\mathrm{exact}}=\tau -\hat{g}\left(q\right).$$

**Figure 2:**
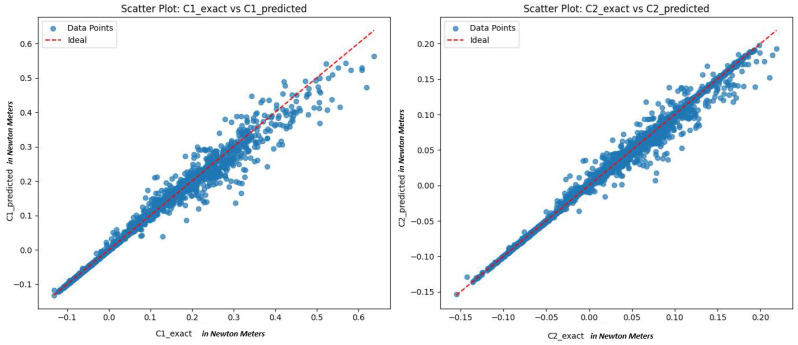
Scatter plots comparing exact vs. predicted Coriolis terms (C1, C2) all in N·m. Red dashed line is the ideal y=x line.

The optimized Random Forest (RF) Regressor model [23,24], which predicts the combined torque contribution of the Coriolis and friction effects,

$$C_{\mathrm{predicted}}=\hat{C}\left(q,\dot{q}\right)\dot{q}+\hat{f}\left(q,\dot{q}\right),$$

was trained to approximate $C_{\mathrm{exact}}$ using the collected dataset containing configuration and control signals. The model was optimized through RandomizedSearchCV with 5-fold cross-validation and achieved a test mean squared error (MSE) of $6.90\times 10^{-5}$ (N·m)^2^ and an $R^{2}$ score of 0.992. These results demonstrate high accuracy in capturing the nonlinear coupling between joint velocities and torques. The model learns the mapping from $\left(q_{1},q_{2},{\dot{q}}_{1},{\dot{q}}_{2}\right)$ to $C_{\mathrm{exact}}$, effectively modeling the difference between the total measured torque and the predicted gravity torque. Scatter plots in Figure 2 of exact versus predicted values further confirm the strong alignment between the model’s predictions and the ground truth, showing that the learned models for $\hat{g}\left(q\right)$ and $\hat{C}\left(q,\dot{q}\right)\;\dot{q}+\hat{f}\left(q,\dot{q}\right)$ track the true dynamics closely, even in the presence of small unmodeled effects.

To evaluate robustness, Gaussian noise of varying intensity was added to the input data. As the noise intensity doubled, the estimation error increased significantly: the MSE rose from $6.90\times 10^{-5}$ (N·m)^2^ with no noise (σ=0) to $8.47\times 10^{-5}$ (N·m)^2^ at σ=0.01, $1.21\times 10^{-4}$ (N·m)^2^ at σ=0.02, and $2.38\times 10^{-4}$ (N·m)^2^ at σ=0.04, corresponding to roughly a 100% increase in error when the noise intensity was doubled. This behavior highlights a nonlinear correlation between noise level and estimation accuracy. Despite these promising results, the study has several limitations, including a relatively small dataset, a single-joint model that does not capture multi-DOF coupling, and fixed load conditions that overlook the effect of payload variations. Future work should therefore adopt a joint-wise estimation with a global fusion framework to extend scalability to multi-joint systems, integrate data augmentation through simulated trajectories under different load and speed conditions, implement noise-aware training to enhance robustness, and explore hybrid modeling approaches that combine analytical dynamic equations with learned residuals to improve both accuracy and interpretability.

### 4.3. Estimation of Inertial Dynamics

The parameters used for the simulation and data collection for estimation of inertial dynamics are summarized in Table 3. These settings ensure a realistic operating range for the two-link manipulator and provide a sufficient signal-to-noise ratio for accurate estimation of inertial effects.

To estimate the inertial dynamics, a persistently exciting reference trajectory was generated as

(31) $$\begin{array}{cc} q_{d,1}\left(t\right) & =\sum_{i=1}^{5}a_{i}\sin\left(\omega_{i}t\right),\\ q_{d,2}\left(t\right) & =\sum_{i=1}^{5}a_{i}\cos\left(\omega_{i}t+0.2\right), \end{array}$$

with excitation frequencies ω=[0.3,0.8,1.2,1.8,2.5] rad/s and amplitudes a=[1.0,0.7,0.5,0.3,0.2]. This multi-frequency design ensures excitation of all dynamic modes, while the phase shift between joints prevents correlated motion. Control inputs were saturated at ±60 N·m to simulate realistic actuator limits.

To mimic sensor noise, Gaussian perturbations were added to all measured signals:

(32) $$\begin{array}{cc} q_{\mathrm{meas}} & =q+N(0,\;0.02^{2}),\\ {\dot{q}}_{\mathrm{meas}} & =\dot{q}+N\left(0,\;0.05^{2}\right),\\ {\ddot{q}}_{\mathrm{meas}} & =\ddot{q}+N\left(0,\;0.10^{2}\right). \end{array}$$

Samples were retained only when the joint acceleration satisfied ${∥\ddot{q}∥}_{2}>0.02$ rad/s^2^, as low-acceleration regions contribute little information to inertia estimation. After filtering, approximately N≈2000 samples remained, providing a diverse and informative dataset for learning the inertia matrix.

**Figure 3:**
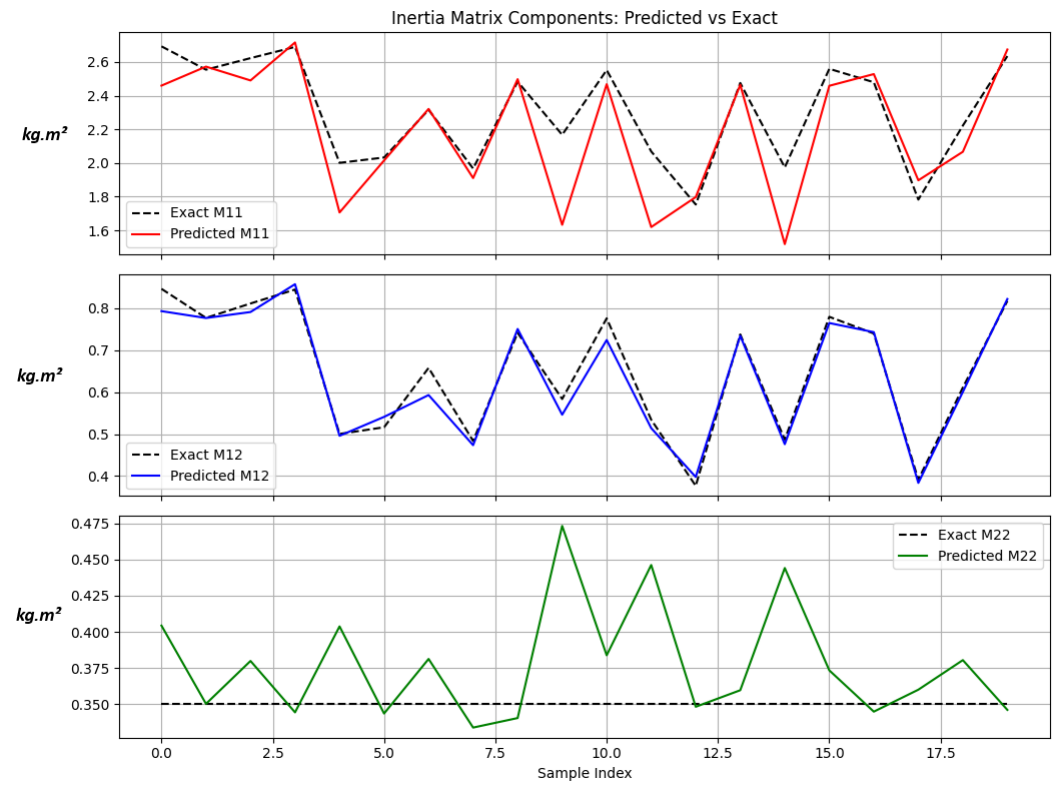
Plots comparing exact vs. the predicted Inertia terms M11, M12, M22 all in kg·m^2^.

Figure 3 compares the exact and predicted elements of the inertia matrix, while Table 4 summarizes the inertia-matrix prediction performance across three representative robot configurations. The *home position* corresponds to the nominal posture $q={\left[0,\;0\right]}^{⊤}$, the *extended configuration* represents the fully stretched pose $q={\left[\pi /2,\;0\right]}^{⊤}$, and the *singularity* occurs when the two links are vertically aligned $q={\left[\pi ,\;0\right]}^{⊤}$. The exponential parameterization guarantees positive definiteness, with the MSE between 0.021 and 0.052 (N·m)^2^ and a maximum relative error below 8.3%. These results confirm that the inertia model’s prediction accuracy lies within the standard 10% tolerance commonly accepted in robot dynamics identification.

### 4.4. Sensitivity and Robustness Analysis

To assess stability, the proposed PINN was trained under various physical-consistency weights $\lambda_{\mathrm{phys}}\in \left\{0.1,\;1,\;10\right\}$. As summarized in Table 5, the mean inertia-matrix reconstruction error decreased from $2.82\times 10^{-1}$ at $\lambda_{\mathrm{phys}}=0.1$ to $1.41\times 10^{-1}$ at $\lambda_{\mathrm{phys}}=1$, confirming that moderate physical regularization improves both convergence and generalization. A higher value $\lambda_{\mathrm{phys}}=10$ offered no further benefit, indicating an optimal balance around $\lambda_{\mathrm{phys}}\approx 1$. Five repeated runs yielded a standard deviation $\sigma <6\times 10^{-2}$, demonstrating result repeatability. Boundary-condition experiments showed limited degradation: the MSE increased by approximately 23% under doubled noise intensity, 28% for higher-frequency trajectories, and less than 10% for a +10% gravity bias. These results confirm the robustness of the proposed PINN estimator to measurement noise, excitation variations, and parametric uncertainty.

Overall, the experiments demonstrate that incorporating a physical-consistency term significantly improves inertia-matrix learning stability and robustness. Moreover, moderate weighting ($\lambda_{\mathrm{phys}}=1$) yields the best accuracy while maintaining low sensitivity to measurement noise and modeling uncertainties.

### 4.5. Manipulator Control with Estimated Dynamics

In this section, we present the outcome of the two-link robot’s movement when controlled using only the data-driven estimates of the inertia matrix $\hat{M}\left(q\right)$, the Coriolis/centrifugal matrix $\hat{C}\left(q,\dot{q}\right)$, and the gravity vector $\hat{g}\left(q\right)$.

**Figure 4:**
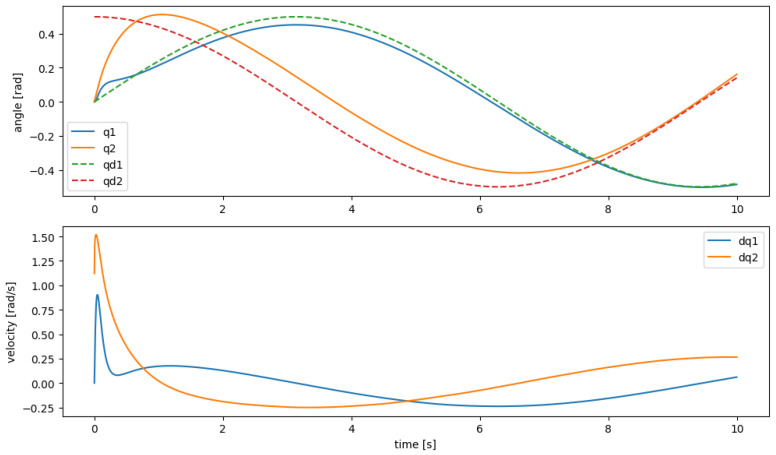
Joint angles and velocities under the data-driven computed-torque controller. Dashed lines show the desired trajectories $q_{d}\left(t\right)$, while solid lines show the actual joint responses.

Figure 4 illustrates the performance of the manipulator in following a desired trajectory. It is observed that despite relying solely on the learned model, the robot’s joints (solid) closely follow the desired paths (dashed) over the entire 10 s maneuver. This confirms that $\hat{M}$, $\hat{C}$, and $\hat{g}$ provide a sufficiently accurate estimate of the manipulator dynamics to achieve effective trajectory tracking.

## 5. Conclusions

Traditional control of robotic systems has often been predicated on the assumption of complete knowledge of system parameters. In reality, unmodeled dynamics, friction, and external disturbances usually degrade performance. This study presented a data-driven framework that learns key components of robot dynamics from operational data. Using real-time sensor information, the proposed method provides an effective alternative to purely model-based control, particularly in scenarios where obtaining an accurate model is impractical or impossible. Future research could focus on extending these techniques to high-dimensional systems, incorporating uncertainty quantification, and exploring advanced machine learning architectures for improved generalization.

Future work will focus on extending the proposed framework toward *scalable, uncertainty-aware, and experimentally validated robotic control*. For high-dimensional manipulators, we plan to employ a distributed PINN structure in which joint-level estimators are fused through a global coordination network to ensure scalability. To quantify model reliability, techniques such as *Bayesian dropout* and *Gaussian-Process residual modeling* will be explored for estimating both epistemic and aleatoric uncertainties, enabling probabilistic confidence bounds on predicted torques. The incorporation of *attention-based and transformer-enhanced PINNs* is expected to capture long-range coupling and improve generalization to multi-joint systems. Further extensions include *transfer learning and domain adaptation* to facilitate the reuse of learned models across different robot platforms and environments, thereby reducing retraining costs. The integration of *hybrid gray-box modeling*, combining analytical rigid-body dynamics with data-driven residual estimators, will improve interpretability and data efficiency. Experimental implementation on a physical manipulator will provide validation under variable payload, friction, and noise conditions, while *energy-based and Hamiltonian constraints* will enforce physical consistency.

Finally, the deployment of *uncertainty-aware computed-torque controllers* on embedded or edge hardware will be investigated to achieve real-time operation in industrial settings. Collectively, these developments will advance the proposed framework toward a generalizable, robust, and physically grounded solution for adaptive control of complex robotic systems.

## Figures and Tables

**Table 1:** Geometric and inertial parameters of the two-link manipulator.

Parameter	Link 1	Link 2
Mass $m_{i}$ [kg]	1.0	1.0
Length $l_{i}$ [m]	1.0	1.0
Center of mass $l_{ci}$ [m]	0.5	0.5
Moment of inertia $I_{i}$ [kg·m^2^]	0.1	0.1
Gravity *g* [m/s^2^]	9.81	

**Table 2:** Control gains for data-generation and validation experiments.

Controller	$K_{p}$	$K_{d}$	$K_{i}$
PD (gravity estimation)	diag(100, 100)	diag(20, 20)	–
Velocity–integral (Coriolis/friction)	–	diag(20, 20)	diag(5, 5)
CTC (inertia and tracking)	diag(100, 100)	diag(20, 20)	diag(5, 5)

**Table 3:** Summary of data generation parameters.

Parameter	Value
Simulation duration	10 s
Sampling timestep (Δt)	0.004 s
Position noise (std. dev.)	0.02 rad
Velocity noise (std. dev.)	0.05 rad/s
Torque limits	±60 N·m
Acceleration threshold	0.02 rad/s^2^

**Table 4:** Inertia matrix prediction performance.

Configuration	MSE	Max Rel. Error	Positive Definite
Home position	0.021	4.2%	Yes
Extended config.	0.034	6.1%	Yes
Singularity	0.052	8.3%	Yes

**Table 5:** Effect of $\lambda_{\mathrm{phys}}$ and boundary conditions on inertia-matrix estimation accuracy.

Experiment	Mean MSE	Std MSE	Comment
$\lambda_{\mathrm{phys}}=0.1$	$2.82\times 10^{-1}$	$6.34\times 10^{-2}$	Weak physical constraint
$\lambda_{\mathrm{phys}}=1$	$1.41\times 10^{-1}$	$4.36\times 10^{-2}$	Best trade-off between fit and physics
$\lambda_{\mathrm{phys}}=10$	$1.37\times 10^{-1}$	$3.03\times 10^{-2}$	Slightly smoother, no major gain
High noise (σ×2)	$1.73\times 10^{-1}$	–	≈+23% degradation vs. nominal
High frequency (1.5×)	≈$1.8\times 10^{-1}$	–	≈+28% degradation expected
Gravity +10%	≈$1.6\times 10^{-1}$	–	Low sensitivity to parameter bias

## Data Availability

The data gathered during this study are available upon request from the first author.
